# Nature-based social interventions to address loneliness among vulnerable populations: a common study protocol for three related randomized controlled trials in Barcelona, Helsinki, and Prague within the RECETAS European project

**DOI:** 10.1186/s12889-023-17547-x

**Published:** 2024-01-13

**Authors:** Laura Coll-Planas, Aina Carbó-Cardeña, Anu Jansson, Vladimira Dostálová, Alzbeta Bartova, Laura Rautiainen, Annika Kolster, Montse Masó-Aguado, Laia Briones-Buixassa, Sergi Blancafort-Alias, Marta Roqué-Figuls, Ashby Lavelle Sachs, Cristina Casajuana, Uwe Siebert, Ursula Rochau, Sibylle Puntscher, Iva Holmerová, Kaisu H. Pitkala, Jill S. Litt

**Affiliations:** 1 Research group on Methodology, Methods, Models and Outcomes of Health and Social Sciences (M3O). Faculty of Health Sciences and Welfare. Centre for Health and Social Care Research (CESS), University of Vic-Central University of Catalonia (UVic-UCC). Institute for Research and Innovation in Life Sciences and Health in Central Catalonia (IRIS-CC), Vic, Spain; 2 Department of General Practice, University of Helsinki, PO BOX 20, 00014 Helsinki, Finland; 3 Helsinki University Hospital, Unit of Primary Health Care, Helsinki, Finland; 4 Charles University, Faculty of Humanities – Centre of Expertise in Longevity and Long-Term Care, Pátkova 2137/5, 182 00 Prague, Czech Republic; 5 Innovation in Mental Health and Social Wellbeing Research group (ISAMBES), Faculty of Health Sciences and Welfare. Centre for Health and Social Care Research (CESS), University of Vic-Central University of Catalonia (UVic-UCC). Institute for Research and Innovation in Life Sciences and Health in Central Catalonia (IRIS-CC), Vic, Spain; 6 Fundació Salut i Envelliment UAB, Casa Convalescència UAB C/ Sant Antoni M. Claret, 171, 4a planta, Barcelona, Spain; 7 Barcelona Institute for Global Health (ISGlobal), Barcelona Biomedical Research Park (PRBB) Doctor Aiguader, 88 08003 Barcelona, Spain; 8 CIBER Epidemiología y Salud Pública (CIBERESP), Madrid, Spain; 9 Universitat Pompeu Fabra (UPF), Barcelona, Spain; 10 Subdirecció General d’Addiccions, VIH, ITS i Hepatitis Víriques. Agència de Salut Pública de Catalunya, Carrer de Roc Boronat, 81-95, 08005 Barcelona, Spain; 11 Institute of Public Health, Medical Decision Making and Health Technology Assessment, Department of Public Health, Health Services Research and Health Technology Assessment, UMIT TIROL – University for Health Sciences and Technology, Hall in Tirol, Austria; 12 Western Uusimaa Wellbeing Services, Health Services, Espoo, Finland; 13 Center for Health Decision Science, Departments of Epidemiology and Health Policy & Management, Harvard T. H. Chan School of Public Health, Boston, MA USA; 14 Institute for Technology Assessment, Department of Radiology, Harvard Medical School, Massachusetts General Hospital, Boston, MA USA

**Keywords:** Psychosocial group intervention, Social connectedness, Loneliness, Psychosocial processes, Randomized controlled trials, Effectiveness, Group intervention, Nature-based social prescribing, Health-related quality-of-life

## Abstract

**Background:**

The negative effects of loneliness on population health and wellbeing requires interventions that transcend the medical system and leverage social, cultural, and public health system resources. Group-based social interventions are a potential method to alleviate loneliness. Moreover, nature, as part of our social and health infrastructure, may be an important part of the solutions that are needed to address loneliness. The RECETAS European project H2020 (Re-imagining Environments for Connection and Engagement: Testing Actions for Social Prescribing in Natural Spaces) is an international research project aiming to develop and test the effectiveness of nature-based social interventions to reduce loneliness and increase health-related quality of life.

**Methods:**

This article describes the three related randomized controlled trials (RCTs) that will be implemented: the RECETAS-BCN Trial in Barcelona (Spain) is targeting people 18+ from low socio-economic urban areas; the RECETAS-PRG Trial in Prague (Czech Republic) is addressing community-dwelling older adults over 60 years of age, and the RECETAS-HLSNK trial is reaching older people in assisted living facilities. Each trial will recruit 316 adults suffering from loneliness at least sometimes and randomize them to nature-based social interventions called “Friends in Nature” or to the control group. “Friends in Nature” uses modifications of the “Circle of Friends” methodology based on group processes of peer support and empowerment but including activities in nature. Participants will be assessed at baseline, at post-intervention (3 months), and at 6- and 12-month follow-up after baseline. Primary outcomes are the health-related quality-of-life according to 15D measure and The De Jong Gierveld 11-item loneliness scale. Secondary outcomes are health and psychosocial variables tailored to the specific target population. Nature exposure will be collected throughout the intervention period. Process evaluation will explore context, implementation, and mechanism of impact. Additionally, health economic evaluations will be performed.

**Discussion:**

The three RECETAS trials will explore the effectiveness of nature-based social interventions among lonely people from various ages, social, economic, and cultural backgrounds. RECETAS meets the growing need of solid evidence for programs addressing loneliness by harnessing the beneficial impact of nature on enhancing wellbeing and social connections.

**Trial registration:**

Barcelona (Spain) trial: ClinicalTrials.gov, ID: NCT05488496. Registered 29 July 2022.

Prague (Czech Republic) trial: ClinicalTrials.gov, ID: NCT05522140. Registered August 25, 2022.

Helsinki (Finland) trial: ClinicalTrials.gov, ID: NCT05507684. Registered August 12, 2022.

**Supplementary Information:**

The online version contains supplementary material available at 10.1186/s12889-023-17547-x.

## Background

Loneliness refers to a negative subjective feeling state of being alone, separate or apart from others, and has been conceptualized as an imbalance or discrepancy between desired social contacts and actual social contacts [1]. This discrepancy leads to the negative experience of feeling lonely and/or the distress of feeling socially isolated even when surrounded by family, friends, or other people. This definition underlines that feeling lonely does not necessarily mean being alone nor does being alone necessarily mean feeling lonely. Indeed, one can feel lonely in the crowd [2, 3].

Three dimensions of loneliness have been described: social, emotional, and existential loneliness [3]. Social loneliness refers to the perceived absence of quality friendships or family connections, i.e., connections within one’s relational space. The term emotional loneliness refers to the perceived absence of someone significant, a person on whom one can rely for emotional support during crises, who provides mutual help, and who affirms one’s value as a person [2]. Existential loneliness differs from social and emotional loneliness. While social and emotional loneliness are associated with a lack of meaningful social relationships and social companionship, existential loneliness is the result of a broader separation related to the nature of existence and, to the lack of meaning in life. Accordingly, an individual may be in the desired company of others but experience existential loneliness [4].

Understanding of the negative effects of loneliness on health and wellbeing has raised awareness at the societal and public health level. In the long–term, loneliness can lead to or aggravate chronic diseases such as cardiovascular disease, diabetes type 2, cerebrovascular disease, as well as anxiety, depression, cognitive and mental deterioration, disability and increase mortality [4–7].

Several studies have shown that social support interventions and regular small group meetings in which members actively participate are among the most effective interventions for alleviating loneliness [8–10]. Specifically, the intervention strategy “Circle of Friends”, a group-based approach of peer support and empowerment developed and led by the Finnish Association for the Welfare of Older Adults, has been shown to be effective to improve well-being and health of lonely older people [11–14].

Experiences and contact with nature can facilitate dynamic processes of social or interpersonal interaction [15, 16] as well as improve aspects of physical and mental health [17]. Various green space designs and nature experiences can deliver diverse benefits with respect to wellbeing. For example, higher levels of species diversity in parks have been shown to improve mental wellbeing [18]; different sensory experiences such as sounds, smells and tactile sensations have a variety of pathways to wellbeing [19] and the participant experience can also affect wellbeing in multiple ways, from adventure-based activities to seated relaxation [20]. When combining contact with nature with regular small group meetings, social processes are reinforced by shared learning, relatedness, and social participation [21, 22]. Accordingly, the social connection experienced by spending time outdoors with others is increasingly being studied to reduce stress, promote cognitive development and to alleviate loneliness [17, 23–26].

Social prescribing is a referral system to connect people with diverse needs with assets in their communities [17, 27]. This emerging socially oriented practice fosters and maintains social connections and, consequently, reduces the risk of social isolation and loneliness and promotes health and well-being. It has also been shown to reduce the number of primary care visits and the use of other health services [12, 28–30]. In the frame of social prescribing, nature-based social interventions offer a novel socio-environmental innovation to improve wellbeing by linking people in need to local natural resources [26, 29, 31].

The European Commission funded project entitled “Reimagining Environments for Connection and Engagement: Testing Actions for Social Prescribing in Natural Spaces (RECETAS)” was launched in March 2021 [25]. The premise of the project is that social prescribing in natural spaces can serve to alleviate loneliness by engaging people in socially organized activities that are connected to the natural environment in which they live and carry out their daily activities [25, 32]. Interventions that reach and engage diverse populations vulnerable to loneliness and who may face barriers to accessing and enjoying public space and outdoor activities in groups will be developed and tested. Importantly, the intervention tested in RECETAS will link nature-based solutions and green infrastructure with professionals working in local health and social care systems. This will strengthen the evidence for causal relationships between experiences in nature, loneliness alleviation, and increase in health-related quality of life.

## Methods

The study protocol has been developed based on the Standard Protocol Items: Recommendations for Interventional Trials (SPIRIT) guidelines [33].

The aim of the three related studies presented in this paper is to evaluate whether the nature-based social intervention “Friends in Nature” (FIN) is more effective improving health-related quality of life and reducing loneliness among people suffering from loneliness than recommending nature-based activities in addition to usual care. Moreover, these studies are also aimed at characterizing the context, including the natural environment in which the sessions take place, understanding the implementation process and the mechanisms linking the intervention and its components to wellbeing benefits, as well as exploring the perceived effects.

### Study design

Three related randomized controlled trials will be implemented under the umbrella of the RECETAS project: the RECETAS-BCN Trial in Barcelona (Spain), the RECETAS-PRG Trial in Prague (Czech Republic) and the RECETAS-HLSNK trial in Helsinki (Finland).

These three trials are designed following a common protocol, share the objectives and approach, and apply the same intervention framework. Notwithstanding, they test the hypothesis in different populations and cultural contexts, and, therefore, the RECETAS intervention and assessments are adapted to the local context and target populations. Accordingly, the trials will be conducted and analyzed separately as independent studies but, in addition, results may also be combined. A qualitative study is nested in each trial and further explained in the process evaluation.

An initial feasibility study was conducted with the objectives of assessing the practicability of recruiting participants, the ability to carry out the study procedures, the implementation of the intervention, and the evaluation of the measurement tools. The feasibility study uses qualitative and quantitative methods and was conducted between March and December 2022.

### Participants

#### Recruitment

The recruitment pathways differ across the trials to reach their specific target population.

The RECETAS-BCN trial will be conducted in Barcelona province and the recruitment process will involve the engagement of and commitment from local organizations. We will use a neighborhood-based participatory approach [34]. Participants will be recruited from primary health and social care settings, third-sector organizations, community groups, and volunteer organizations who will identify potential participants.

The RECETAS-PRG trial will be conducted in the city of Prague, and it will be focused on older persons living at home who will be reached via different information channels: leaflets distributed to GPs and care providers, contact with senior’s organizations, and information in media (radio, newspapers).

The RECETAS-HLSNK trial will be conducted in Helsinki and metropolitan area and the participants will be recruited directly from 25 assisted living facilities by interviewing all residents who are cognitively able and willing to answer a screening questionnaire.

#### Eligibility criteria

A common eligibility criterion of the three trials is suffering from loneliness according to a screening question tailored to the cultural context as following: “Do you suffer from loneliness?” in Barcelona and Helsinki and “Do you feel lonely?” in Prague. Participants would screen positive when answering ‘sometimes’ or ‘often, or always’ but not if they say, ‘never or hardly ever’ [12].

In the three trials, those having a serious illness with a prognosis of less than 6 months will be excluded. In Barcelona and Prague, participants will be excluded if they have: any disability (i.e., mental, cognitive, somatic, or sensorial), cognitive decline or any mental health disorders in case it prevents them from participating in the group dynamics and activities in nature or it might interfere with the social interactions.

The RECETAS-BCN trial will focus on adults (18+) from socio-economically deprived areas. Specific eligibility criteria in Barcelona are being 18 years or older; being able to give informed consent in Spanish or Catalan; being able to participate in group dynamics and communicate in one of the local languages, to have capacity to walk independently and to be willing to undergo study measurements. Groups will include participants from the same local area.

The RECETAS-PRG trial will include community-dwelling older adults. Specific eligibility criteria in Prague requires the participant to be over 60 years old, living in the community, understanding the informed consent in Czech and to be willing to undergo study measurement.

The RECETAS-HLSNK trial will include older adults living in assisted living facilities. Residents identified by staff will be approached to respond to a screening questionnaire by an interview. The questionnaire includes questions about loneliness, wishes for nature-based experiences and willingness to participate in group-based intervention. Specific eligibility criteria in Helsinki require the participant to be at least 55 years old, to live permanently in assisted living facility, participate in the study voluntarily, to have the Mini-mental State Examination (MMSE) at least 15 points (i.e., not being moderately-severely cognitively impaired), to be able to move with or without assisting devices and to have sufficient sight, hearing, and communication skills to participate in group activities.

In the three trials, if an individual meets the eligibility requirements, the study personnel will explain the intervention and the randomization procedures, data collection requirements as well as the risks and benefits of participating in the trial. Trial information will be offered in local languages and informed consent from participants will be collected before the baseline assessment.

#### Sample size

The sample size has been calculated to detect a clinically significant difference of 0.015 - 0.04 in the primary outcome HRQOL-15D [35] between the intervention and control arms. The calculation is based on a typical standard deviation in this type of population 0.11, type I error 5% and power 80%. The sample assumes a 25% loss to follow-up. The sample size calculation assumes that participants in each study site will be analyzed independently. The sample size was evaluated using simulation-based sample size calculation. The resulting sample size is 316 participants per city (158 per randomization arm). The group-based intervention requires 5-12 persons per group, with means building approximately 13-30 groups in each city to reach the 158 participants allocated to the intervention arm.

#### Randomization

Each trial will conduct a centralized randomization procedure. Computer-generated random allocation sequences will be generated for each trial, using blocks of varying sizes. The allocation sequence is generated by a researcher who is not involved in the data collection process. A different researcher enrolls participants and assigns them to the corresponding arm. In addition, the allocation to the study conditions is securely stored. Cohabiting couples will be randomized to the same study group, to avoid contamination. Concealed allocation of participants to intervention or control arm will be conducted after baseline assessment, being participants informed of their group allocation at this timepoint.

As a strategy to reduce attrition, study staff will discuss study expectations with participants prior to randomization and will ask eligible participants whether they will be able to commit to the study protocol. Individuals who do not feel that they can maintain this commitment will be excluded from the study, while those positive about their commitment to the study protocol will be enrolled. Likewise, as a strategy to increase the retention in the study after randomization, study personnel will maintain contact with intervention and control participants through assessment time points and interim check-ins to minimize dropout and loss to follow-up.

#### Blinding

The study will conduct blinded outcome assessments. The professionals informing participants of their allocation and those delivering the interventions will be different from outcome assessors. Baseline questionnaires and measures will be conducted at T0 before random allocation to ensure blinding. When conducting the rest of assessments, outcome assessors will ask participants not to disclose their allocation during their interactions. However, due to the nature of the intervention, it might be difficult to avoid participants providing information that could help assessors to know their assigned arm. Therefore, in Barcelona, to assess the success of this strategy, outcome assessors will report after each assessment whether participants revealed their allocation, or if they could guess it.

The delivery of the intervention cannot be blinded due to its nature and participants are aware of being part of the intervention or the control arm. Data analysts will be blinded to participant allocations.

### Intervention arm: “Friends in nature” (FIN)

FIN is described based on The Template for Intervention Description and Replication (TIDR) guidelines [36].

**Fig. 1:**
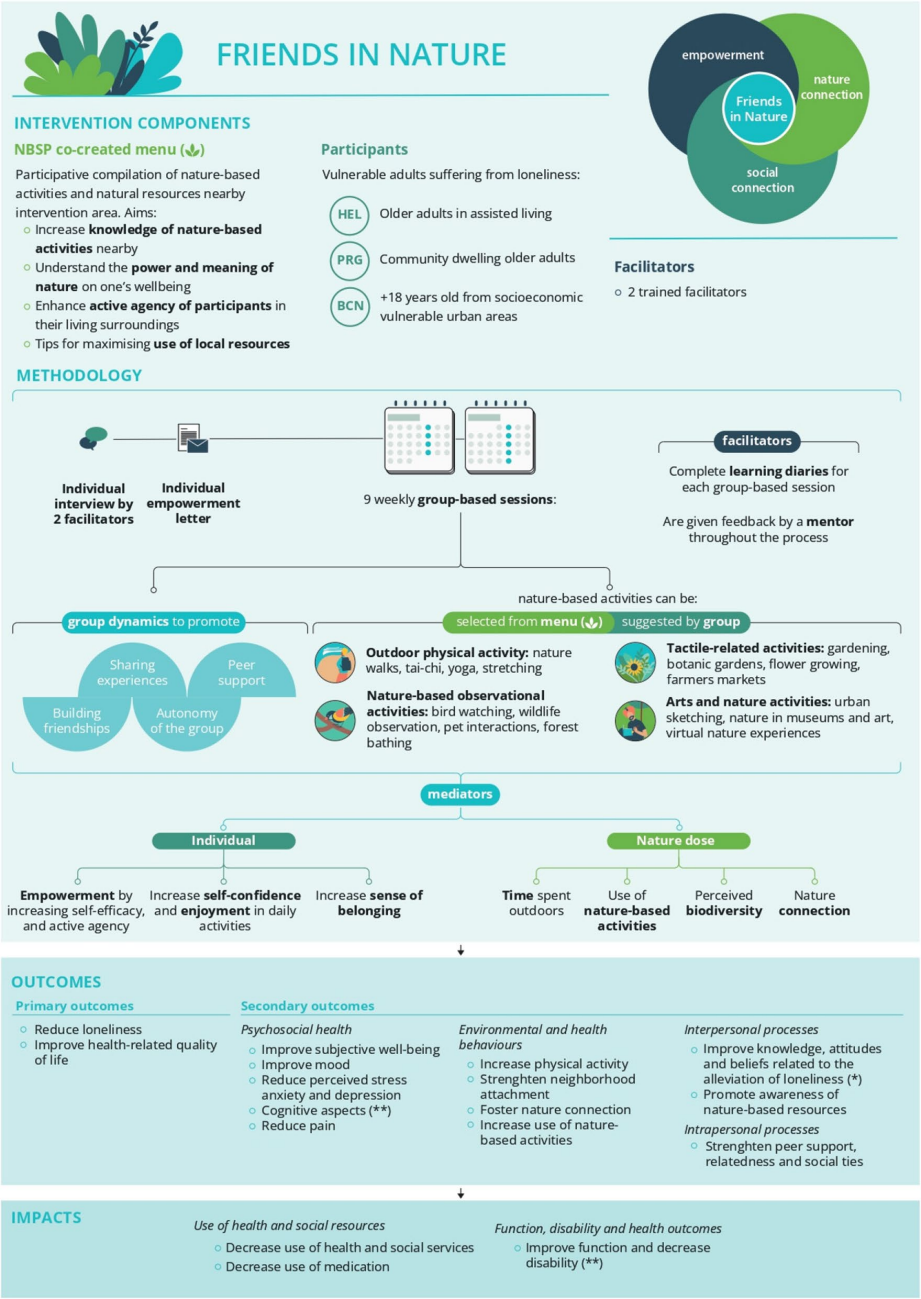
Explanation of the intervention process for the three trials of the RECETAS project

FIN is an adaptation from the Circle of Friends® methodology customized to the specific target population in each trial and with a focus on nature-based activities. This complex intervention has two main components that are expected to complement and make synergies with each other: 1) peer support group and empowerment process including specific group dynamics and elements that were adapted according to the Circle Of Friends® methodology (individual interview, empowerment letter, diaries and training) [14] and 2) the nature-based activities chosen by participants from a menu based on their preferences. Figure 1 provides a schematic explanation of the intervention model. Trained facilitators are key persons in the intervention, their training is described in section 2.3.1.

The intervention requires 5-12 persons per group. Two trained facilitators are assigned to each group to support the group dynamics by fostering empowerment and, at the end of the group process, independence from the facilitator. Two facilitators enable the study team to observe the group more thoroughly, give feedback to each other, and make better use of group dynamics, as well as increasing safety.

#### Co-created menu

This menu describes the nature-based activities and resources in the intervention area and can help participants to increase their knowledge of these opportunities nearby and provide tips for maximizing their use. The menu includes activities promoted by the municipality or grassroots organizations which can accommodate the group of participants, open and freely accessible nature areas, or new activities specifically organized for the RECETAS group (e.g., urban sketching).

To develop the menu, local stakeholders were engaged in a social network analysis in the earlier stages of the RECETAS project. The co-creation process enabled the development of this menu, which is tailored to the local resources. The co-creation process conducted for the RECETAS-BCN trial is explained in a separate paper [34].

#### Individual interview

During week 1, participants assigned to the intervention arm will undergo an individual interview by the pair of trained facilitators. The aim of the interview is to identify individual’s expectations, nature-based interests, and achievement goals regarding social connections and loneliness alleviation and to create an environment that allows participants to experience they are being heard and seen. This discussion is an important first step in connecting and building trust with the facilitators, prepares the individual for the group process and enhances the opportunities to influence one’s own life situation. Additionally, this individual consultation allows the participants to develop personalized plans to strengthen social connections and improve wellbeing, and to articulate their feelings about being in nature and the kinds of activities they enjoy, are willing to try, and/or concerns about being outdoors or part of a group. Finally, the interview also helps the facilitator learn about the life of the participant and plan the start of the group process.

#### Empowerment letter

After the interview, facilitators write a personally tailored Empowerment Letter for each participant which is delivered before the first day of the session. This letter supports the participant’s empowerment and highlights the person’s strengths and topics that are important to them in their everyday life as they emerged in the interview. The letter is also aimed at encouraging them to take part in the first group session, to start with enthusiasm and to commit to continuing.

#### Group-based sessions: empowerment, social connectiveness and connection to nature

The 5-12 participants allocated to intervention group are invited to join nine group-based sessions once a week with a duration of at least 2 hours after the individual interview. On one hand, as proposed by Circle of Friends® methodology, group sessions are aimed at building supportive relationships, developing new social ties, and learning about ways in which they can alleviate loneliness. On the other hand, as added in the RECETAS project, groups also aim to increase the understanding of the power and meaning of nature on one’s wellbeing, enhancing active agency of the participants in their living surroundings and increasing time spent outdoors with nature. The social and the natural component of the intervention are expected to act synergistically with each other reinforcing their effects.

The social component of the intervention is organized around several key activities. Group dynamics include learning to know each other (for example, through a personal object), or establishing ground rules for a positive group environment. Discussions and activities include both loneliness, and how to alleviate it, and nature as a source of wellbeing, as well as dynamics to foster group cohesion around cultural aspects such as food, music, dance, and so forth. These activities might be carried out both indoors and outdoors, but priority is given to activities outdoors in natural environments, weather permitting. To enhance the nature component, the menu is introduced during the first group session and used to show available nature-based activities. Moreover, the group will share their level of experience in nature, and their respective interests, hobbies, and preferences, so that the group together can choose and plan the activities that they want to explore, based on the menu and their own ideas. Activities in nature can be classified as: engaging with nature (e.g., gardening, planting, bird watching, forest bathing), being in nature (e.g., walking) and social activities done in natural surroundings (e.g., discussion about loneliness, picnic, music). Through the group process, participants will increase their social and peer support and emotional wellbeing as they participate in socially supported nature-based activities. As the intervention progresses, facilitators gradually step back, and the group plans how to continue group meetings and maintain connections after intervention ends.

#### Learning diaries

Learning diaries are written together by the two trained facilitators to capture group processes through written observation after each session describing the main aims for the specific session, the development of the session and the aims for the next session. Reporting participant experiences promotes reflective learning and helps evaluate the goals of the group meetings with the co-facilitator.

#### Training and mentoring facilitators

The training of facilitators is central to the RECETAS interventions. There, facilitators will learn about loneliness, the elements of the original Circle of Friends® group model [37], how to plan a group, how to conduct the interview and write the empowerment letter, and how to facilitate the group processes and dynamics of FIN. The training also provides content on how to include nature-based activities within the group dynamics, why and how contact with nature helps improve mental and physical health, and social connectedness and might alleviate loneliness. The training combines theoretical sessions with reflections, dynamics, and feedback. Based on these experiences and feedback received by trainer and peers, facilitators can form their own integrated knowledge based on theory, personal experience, and active reflection.

The facilitator training program will be adapted from the Circle of Friends® methodology in each city for the corresponding trial [37]. Initially, the team from Finland from the Finnish Association for the Welfare of Older Adults will conduct a series of webinars to train the trainers in Barcelona and Prague. Those trainers will facilitate the pilot intervention while being monitored and mentored by the Finnish team to finalize their training. These facilitators will then become the new local trainers in charge of educating and mentoring new local facilitators conducting the intervention groups during the trial.

Mentoring is also an important part of the training process and consists of reading the empowerment letters and the weekly learning diaries and providing periodic feedback to facilitators. Accordingly, the mentor fosters a process of reflection, evaluation, and feedback and, thus, promotes growth in the group facilitators roles. The training process also includes observations by trainers at selected moments in the intervention to observe group processes. These observations are shared with the group facilitators and discussed to ensure the group process continues in line with the scope of the intervention [37].

### Control arm

In Barcelona and Prague participants of the control arm receive a brief intervention consisting of signposting, i.e., a professional provides them individually information and choices to participate in local nature-based activities available in the co-created menu. The menu is printed and delivered to control participants as a resource sheet or leaflet and explained to the participants during an interview. In addition, these participants will further receive standard care, including social prescribing from primary health care and social care if available.

In Helsinki, as participants are dependent on the staff, both residents (participants) and relatives receive information about the trial and the favorable effects of nature in a common meeting but otherwise receive usual care.

### Outcomes

**Fig. 2:**
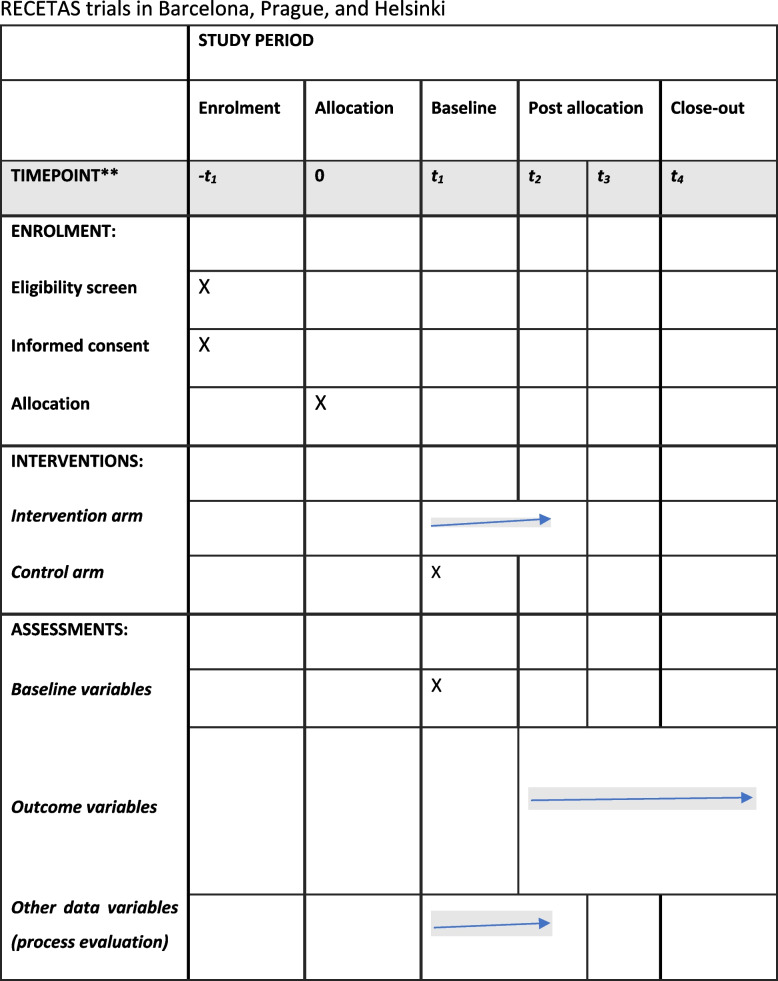
SPIRIT 2013 schedule of enrolment, interventions, and assessments for the RECETAS trials in Barcelona, Prague, and Helsinki

Outcomes will be assessed at baseline (T0), month 3 (T1, end of the intervention), month 6 (T2, 3 months post the intervention) and month 12 (T3, 9 months post the intervention). All researchers in charge of conducting the assessments will undergo a training session. These elements are described in Fig. 2, in accordance with the SPIRIT 2013 trial guidelines.

At baseline, socio-demographic information regarding age, gender, educational level, living arrangement and working situation will be collected.

Primary outcomes of the three trials include: health-related quality of life measured with the HRQOL-15 D questionnaire [35] and overall loneliness assessed by The De Jong Gierveld 11-item loneliness scale, which also measures separately social and emotional loneliness [38].

**Fig. 3:**
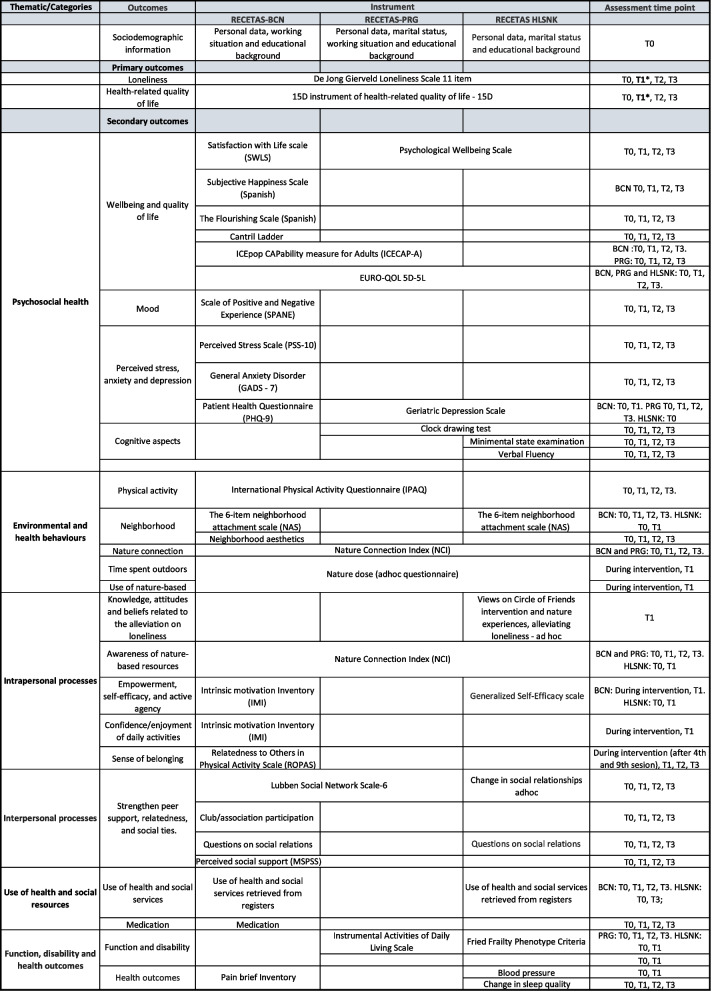
Primary and secondary outcome measures, instruments, and assessment time points for the RECETAS trials for Barcelona, Prague, and Helsinki. Footer: *Primary outcomes are considered at post-intervention (T1), T2 and T3 are assessed as secondary outcomes; T0: Baseline assessment; T1: 3 months from baseline (post intervention); T2: 6 months from baseline (3 months after the end of the intervention); T3: 12 months from baseline (9 months after the end of the intervention)

Secondary outcomes will vary according to the study and population and will measure changes in psychosocial health (e.g., subjective well-being, quality of life, utilities, capabilities, mood, perceived stress, quality of sleep, anxiety, and depressive symptoms, and cognitive aspects); environmental and health behaviors (e.g., physical activity, time spent outdoors, and use of nature-based activities); intrapersonal processes (e.g., knowledge, attitudes, and beliefs related to the alleviation of loneliness; awareness and use of nature-based activities); interpersonal processes (e.g., peer support, relatedness and social ties, social involvement); use of health and social resources (use of health and social services and medication) and their corresponding costs as well as the costs of the intervention itself; and function, disability and health outcomes. For an overview of primary and secondary outcomes specified in each city, outcome measures, instruments, and assessment time points, see Fig. 3.

Intermediate factors or mediators of the impact will be measured with standardized scales during the intervention. These indicators include empowerment by increasing self-efficacy and active agency; increasing confidence, relatedness, and enjoyment; and increasing sense of belonging (Fig. 3). Moreover, a health economic questionnaire has been developed to assess the amount of health and social services used (including medical consultations, in-patient hospital services, medication, or community care contacts).

To evaluate nature exposure prompts it is needed to assess their experiences in nature both during the session and the time spent in nature the last week through a short questionnaire. Nature exposure received by participants during the intervention period is assessed as the activities in nature conducted during the sessions. Specifically, we will characterize three components of the nature experience that have previously been linked to mental wellbeing. First, the actual and perceived biodiversity of the green or blue space will be recorded. Actual species diversity will be characterized as the number of species present and their functional characteristics, for example, the species richness and abundance of street trees in a park. Data will be derived from remote sensing and existing surveys of the environment in which the intervention takes place. Perceived biodiversity will be recorded through participant feedback [18, 19]. Second, exposure to nature will be recorded as a measure of the time and/or proximity to nature during the intervention. Third, the type of experience will be measured as either incidental (e.g., observing nature on a walk), or experiential (e.g., planting trees). The evaluation takes place after each session of the intervention group, either by filling out a paper- or digital form, as preferred by participants. In this moment, participants are asked to report also the activities conducted last week outside the sessions. Participants of the intervention arm in the three trials with difficulties in reading or writing will be assisted in filling respective questionnaires about the sessions in nature.

Baseline confounders, such as age, gender, or education, will be measured at the beginning of the trials (T0). For measuring time-dependent confounders as discussed with experts in social science, epidemiology, and causal inference, a short questionnaire on mental and physical wellbeing, self-confidence and the influence of the weather will be collected during the intervention.

In Barcelona and Prague, control group participants will complete a diary with the weekly time spent in nature. Furthermore, in the RECETAS-HLSNK trial, the days and time spent outdoors outside the group will be retrieved from the nurses’ records of the assisted living facilities for each study participant of both arms.

### Process evaluation

Nature-based social interventions are complex and require a process evaluation to understand how implementation, causal mechanisms, and context shape outcomes. Therefore, the three trials comprise a process evaluation designed following the Medical Research Council guidance [39] to assess specifically fidelity and reach of the implementation, the contextual aspects of each intervention site, mechanisms of impact, and perceived effects.

As part of the trial protocol, a quality control protocol will be designed to monitor intervention reach. Specifically, the intervention reach will capture the percentage eligible to the study who choose to enroll, participants who drop out of the intervention and why, and those who complete assessments at each time point. This part of the study will provide important information for future real-life implementation.

Methodologically, mixed research methods, i.e., a combination of qualitative and quantitative procedures, are applied. As quantitative procedures, attendance registries will be used to assess the adherence of each participant to the group-based sessions and fidelity checklists will be applied to measure the degree of implementation of the intervention as planned. Dose delivered (i.e., fidelity) and received (i.e., adherence) will shed light on whether participants attend the intervention, how often they attend, and the activities in which they participate. In addition, standardized scales will capture those variables considered a priori as intermediate factors or mediators of the impact on the outcome variables as commented before. Reported adverse events and other unintended effects of the interventions such as falls and other accidents during the activities and interpersonal conflicts will be recorded in the learning diary, analyzed, and reported.

Qualitative methods will be used to describe participants’ experience of loneliness, explore the processes undergone such as the dynamics in the groups, and elucidate the experiences of the intervention, whether and how they are maintained, and the mechanisms underlying the effects. Likewise, at group level, the dynamics will be analyzed as a key element to understanding the process of each group and how participants use the elements of the FIN intervention.

Several qualitative techniques will be used, and triangulation techniques will provide us complementary views from various angles. Specifically, semi-structured interviews with study participants and professionals, when appropriate, will be conducted, as well as researcher’s participant observations of several group sessions. In addition, we will include facilitators diaries of each group session describing the group processes, as material for the qualitative study. Participants for the qualitative interviews will be selected to reach an heterogenous sample according to the main characteristics affecting process and effects (e.g., age, gender, cultural background, and socio-economic background).

The analysis will be inductive, and the qualitative and quantitative findings will help to refine the theory of the intervention to finally support the interpretation of the results on the effectiveness of the intervention.

### Ethics and dissemination

The study design was approved by the corresponding Ethics and Research Committee of each intervention site: The clinical trial of Barcelona received the approval of the Research Ethics Committee (REC) from UVic-UCC (Code: 214/2022), and the Research Ethics Committee of the Primary Health Care Research Institute of Catalonia Jordi Gol (Code CEIm: 22/170-P). The clinical trial of Helsinki obtained the approval of the Helsinki University Hospital Ethics Committee. They also received approval from Social Services and Health Care of the City of Helsinki. Finally, the protocol in Prague was approved by the Ethics Committee of the Faculty of Humanities, Charles University. Participation is voluntary and all participants (and their closest proxy when appropriate) will be asked to sign informed consent before the start of the study.

Ethical aspects of the studies and arising concerns are carefully followed and discussed during the team meetings. Specifically, RECETAS has defined a steering committee lead by the coordination center (ISGlobal) with representants of all partners who meet monthly for continuous update and decision making of each WorkPackage (WP), including the trials. Moreover, a specific WP is in charge of the ethics requirements with an independent Ethics Advisor. Last, an External Advisory Board periodically oversees the progress of the project and supports decision making on relevant issues.

Regarding the dissemination plan, a publication committee with rotating members has been established to supervise scientific dissemination. The results of the studies will be published in open access regardless of the outcome. Researchers will communicate trials results to participants, professionals involved and stakeholders once data is analyzed after finishing all the studies. Moreover, RECETAS uses social media (Twitter, web page, Instagram, and LinkedIn) to support communication of the results to the general public.

### Data and statistical analysis

#### Analysis plan

All randomized participants will be included in analyses under an intention-to-treat (ITT) approach, where all participants will be analyzed in the group they were originally allocated to if they have at least two measurement time points available, regardless of protocol violations. We will compute statistical comparisons between the groups using t-tests, Mann Whitney U tests, or Chi-Square tests when appropriate. Repeated measures will be analyzed using mixed models, with appropriate distribution and link functions, and an unstructured correlation structure, with treatment groups, time, and their interactions as fixed factors. Incidence rates of health and social services will be estimated and compared between the groups using the Poisson type regression models. A Cox Proportional Hazard model will be used to test whether allocation to intervention or control arm has efficacy on mortality. The normality of the variables will be tested graphically and by using Shapiro-Wilk W tests. All analyses will be adjusted for relevant covariates and effect modifiers (e.g., age, gender, comorbidities). In cases where assumptions are not met (e.g., non-normality) for continuous variables, a bootstrap-type method or Monte Carlo *p*-values (small number of observations) for categorical variables will be used. In addition to the ITT analysis, a causal inference-based per-protocol analysis will be performed to assess the effect of compliance on the outcomes of loneliness and quality of life using a structural nested model with g-estimation [40, 41]. Furthermore, cost-effectiveness analyses along the trials (based on loneliness outcome), cost-utility analyses along the trials and cost-capability analyses along the trials will be performed. Several secondary and subgroup analyses will be performed (e.g., for stage of dementia, type of loneliness, etc.) to identify effect modification.

### Data management and monitoring

A specific WP called “Evaluate Nature-Based Social Prescribing through Intervention Studies”, led by UVic-UCC, coordinates the three trials and, among its tasks, data management and data monitoring are the responsibility of ISGlobal. The RECETAS Data Management Plan has established guidelines to inform how each partner involved in the three trials has to proceed with managing the data. Each participant will have a code and the respective answers associated with that code. All the information will be collected at Redcap (Research Electronic Data Capture), a secure, web-based software designed to support data capture for research studies for the creation and management of online databases and surveys ensuring anonymization [42, 43]. The assessor has restricted the information to ensure participant assignments are blinded.

## Discussion

The three RECETAS trials will provide evidence on the effectiveness of a nature-based social intervention tailored to a diversity of vulnerable populations suffering from loneliness (adults from socio-economic disadvantaged urban areas, older people living in assisted living and community-dwelling older adults) in three different cities (Barcelona, Helsinki, and Prague). Thus, the target populations will comprise a diversity of age groups, languages, socio-economic levels, in different cultural contexts with varying climates, natural resources and community assets within Europe.

In the recruitment process, identifying people suffering from loneliness might be challenging, due to the complexity of this phenomenon. First, loneliness is a subjective feeling that might be difficult to recognize for oneself and to communicate to others. Moreover, it is a dynamic feeling that changes over time [44]. Second, there is stigma and taboo around it and lonely individuals might deny suffering from it. Last, it can also be erroneously identified by professionals referring participants who live alone or have limited socials contacts but do not feel lonely.

Signposting of nature-based activities has been chosen as comparison next to control arm. This low level of social prescribing is based on a brief intervention and works best for people who are confident and skilled enough to find their own way to services [27]. However, we aim to find meaningful differences when compared to the group-based intervention, especially when considering the profile of participants and their condition of suffering from loneliness.

The three-related but independent trials have been designed following a common protocol, sharing the objectives and approach, and applying the same intervention framework. Notwithstanding, the RECETAS intervention and assessments are adapted to the local context and target populations. However, the shared assessments such as the primary outcomes (15D and De Jong Gierveld Loneliness scale) [35, 38] might work better for one or the other population. For instance, the low levels of functional disability expected in the younger population targeted in Barcelona might suggest 15D having a ceiling effect, while they might be sensitive to change with the population of Prague and Helsinki. On the other hand, the process evaluation and the qualitative study nested in each trial will support understanding the specificities and common pathways and mechanisms across the three sites.

It is important to consider the difficulty of maintaining the blinding of outcome evaluations. Although we ask participants not to disclose it, it is very difficult to prevent participants from revealing the group to which they have been assigned or giving any clue, when answering questionnaires about friendships, daily activities, etc. Another challenge is the loss of participants along the study from recruitment to the 12-month assessment, since we target vulnerable population including frail and disabled older population and younger population with socio-economic burden. A further limitation is the restricted time horizon of the trials. To estimate long-term effectiveness and cost effectiveness, a decision-analytic model will be developed.

Interventions on loneliness trying to show effectiveness face several challenges and the FIN is not free of them [45]. With the FIN intervention, we work at group level (meso level) aiming to impact individual’s wellbeing at micro level. However, it does not impact the social determinants of health at macro level such as the living situation and the socio-economic constraints, which are also main drivers of loneliness. FIN offers a range of opportunities to increase social connectedness in quantity and quality and promote participation in nature-based activities and resources in a safe environment of peer support. Accordingly, different profiles of persons with social or emotional loneliness might find their own pathway among these elements to alleviate their suffering. Nevertheless, FIN is not meant to address all forms of loneliness and social needs, but it is a solution that could benefit especially those who like groups and nature.

Results will potentially lead to validation of the effectiveness of Nature-Based Social Prescription in supporting populations at risk of loneliness via engagement in socially oriented opportunities in safe, inclusive, and accessible green and blue outdoor urban spaces [25]. Accordingly, RECETAS meets the growing need for programs addressing loneliness and quality of life by harnessing the beneficial impact of nature on enhancing social connections. The three trials will provide evidence on pathways or mechanisms on how nature (type and dose) influences quality of life.

If successful, the three RECETAS trials will provide an evidence-based approach for using social prescribing to address loneliness. FIN represents a low-cost, creative means to strengthening social networks, reducing stress, and facilitating social connectedness among participants and providers. We believe that investments in FIN, as a nature-based social intervention, will lead to improved urban health and well-being by promoting aesthetic experiences, increasing active citizenship, strengthening neighborhood ties, and fostering social connections across different social and economic groups. This will harness the social processes that are fundamental to sustainable behavior change and that will improve both mental and physical health, as well as the policies needed to maintain and enhance the benefits beyond the scope of the RECETAS project.

### Supplementary Information

**Additional file 1.**

**Additional file 2.**

**Additional file 3.**

**Additional file 4.**

## Data Availability

The RECETAS Data Management Plan has established guidelines to inform which data will be open, available upon request, and restricted to project personnel. All open research data in RECETAS will be deposited in a certified repository (e.g., ZENODO) and open access will be established to identify users for access and use of this data. Other resources and tools developed in the project will be made available via the project website (www.recetasproject.eu) or upon request. Data sharing is not applicable to this article as no datasets have been generated thus far.

## Abbreviations

FIN: Friends in Nature
RCTs: Randomized control trials
HRQOL: Health-related quality of life
ITT: Intention-to-treat.
NBS: Nature-based solutions
NBSI: Nature-based social interventions
RECETAS: Reimagining Environments for Connection and Engagement: Testing Actions for Social Prescribing in Natural Spaces
SPIRIT: Standard protocol items: recommendations for interventional trials
REC: Research ethic committee
REDCAP: Research Electronic Data Capture
MMSE: Mini-mental State Examination
WP: Workpackage
