# Supplementary material for: Nature-based social interventions to address loneliness among vulnerable populations: a common study protocol for three related randomized controlled trials in Barcelona, Helsinki, and Prague within the RECETAS European project
Source: BMC Public Health. 2024 Jan 13;24:172. doi: 10.1186/s12889-023-17547-x (PMC10787456; doi:10.1186/s12889-023-17547-x)
Supplement: Supplementary file 1 — Additional file 1. [file 12889_2023_17547_MOESM1_ESM.pdf]

NOTICE OF RESEARCH

Elderly people's experiences of loneliness and the importance of nature in assisted living

Request to participate in research

Your immediate family members are asked to participate in a study that investigates the loneliness of elderly people living in assisted living facilities and the importance of nature experiences for well-being.

This information describes the research and your next of kin's possible participation in it. Feel free to read this announcement. If you have any questions, contact the researcher or other research staff (contact information can be found at the end of the document).

If your next of kin decides to participate in the study, you will be asked to sign a separate consent form on their behalf.

HUS's research ethics committee has issued a favorable opinion on the research plan.

Voluntary participation, suspension and withdrawal of consent

Participation in this study is voluntary. You can deny your next of kin participation in the study, suspend participation, or withdraw already given consent to this study without giving a reason, at any time during the study without affecting your next of kin's right to receive the treatment he needs.

If you wish to cancel your close relative's participation in the study, contact researcher Laura Rautiainen, tel. 050 317 2562,laura.rautiainen@vtkl.fi

You have the right to withdraw your consent at any time - including during the study - before the end of the study, so that the information collected about your next of kin can be used in the study.

What is studied and why

The loneliness of elderly people is a significant challenge in our society, as it can lead to e.g. impaired quality of life, memory impairment,

to a weakened state of health and to the risk of premature death. Experiences of loneliness are common among elderly people in long-term care, more than a third suffer from loneliness at least sometimes. The COVID-19 pandemic has limited the living environment of elderly people and increased loneliness, especially in service homes. However, the effect of nature experiences on the alleviation of loneliness in elderly people has not yet been studied, so research related to the topic is needed.

The purpose of the study is to find out the loneliness experienced by the elderly living in a service home and the importance of nature experiences for their well-being. The study finds out what kind of loneliness the subjects experience in a service home, how the subjects feel that loneliness affects their well-being, and in what ways, in their opinion, loneliness can be prevented, alleviated and reduced through nature experiences. In addition, the research investigates the experiences of the subjects of nature-based group rehabilitation.

The study will include 16-25-year-old people living in Helsinki's care homes who experience loneliness. They are offered group activities based on nature experiences ("Circle of Friends"). The research is carried out in the subjects' own service buildings, in Helsinki, Finland.

The nature-oriented Ystäväpiiri group offered in the study meets in the subjects' own service center once a week, at the same time each week a total of 9 times. The group is led by trained group leaders who take notes on group situations for research. 6–10 elderly people participate in one Ystäväpiiri group. The group's goal is shared nature experiences and through them alleviating loneliness, supporting friendships and a comfortable time together. In the group, we have coffee, carry out nature experiences according to the wishes of the participants and discuss issues of interest to the participants.

How to research

The research is carried out as a qualitative interview and observation study. At the beginning of the study, the background information, well-being and health of the subjects are surveyed using a questionnaire.

Participation in the study takes about six months. The research includes participating in a nature-oriented Ystäväpiiri group for three months.

The research is carried out in such a way that the researcher interviews your next of kin individually 2–4 weeks before the start of the Friends Circle group and 2–4 weeks after.

The researcher visits the Ystäväpiiri group to monitor its progress and makes notes on the observations. During the research, the group visits are recorded. Approximately 3–6 weeks after the end of the Ystäväpiiri group, the researcher organizes one more additional group discussion, where experiences in nature-oriented Ystäväpiiri group activities are discussed. From this group discussion, the researcher takes notes for the research. The interviews conducted during the research are recorded.

If your next of kin is interested in the study, we kindly ask you to fill out the attached form and return it to the nurse in his unit. The researcher will contact you personally in the near future and tell you more about the research. After this, you and your next of kin can voluntarily decide if they want to participate in the study. The researchers will only receive the information about your next of kin that he himself gives during the investigation. This information remains for research use.

Termination of research

After the group discussion that takes place after the end of the circle of friends group, the researcher prepares a report from the material he has collected, in which it is not possible to identify the research subjects. Subjects can read the report after it is completed.

From the start of the research to the publication of its results, it is estimated to last five years as a whole, from which time the portion of the researched is estimated to last about six months.

Conductor and financier of the study

The study is carried out by the University of Helsinki and the Hospital District of Helsinki and Uusimaa. The research takes place in Helsinki serviced apartments or enhanced serviced housing units.

This qualitative study is carried out by researcher Laura Rautiainen as part of her dissertation research. The person responsible for the study is professor emerita Kaisu Pitkälä, who is responsible for the safety of the research subjects. The research commission is the University of Helsinki.

Research costs and financial statements

The University of Helsinki and the EU-funded RECETA Project are responsible for funding the research. The RECETAS project pays the research center compensation for carrying out the research. Research nurses are paid a separate compensation for conducting research.

Possible benefits and risks of research

The potential benefit of the research is that the loneliness of elderly people who participate in the nature-oriented Ystäväpiiri group will be alleviated, their health-related quality of life will improve, their memory will improve, and this will also be reflected in their health and well-being. Subjects are at particular risk of the harms of loneliness and isolation, such as declines in well-being, health and memory, and thus the benefits they receive can be great. Likewise, by participating in research, they have the opportunity to participate in nature experiences that may be particularly well-being enhancing.

Possible disadvantages and inconveniences caused by the study

This study will not cause any harm to your next of kin, nor does it involve special risks or significant discomfort. The content of the nature-oriented Ystäväpiiri group is planned according to the wishes of the elderly people participating in the group and on their terms. If necessary, the study doctor assesses the subjects' condition after the research nurses' examinations and before the start of the group, so that the subjects are not exposed to risks due to their illnesses. If diseases that require a doctor's examination come up during the examination, this will be reported to the service center and the responsible home care doctor.

The safety of the research subjects is guaranteed on excursions with the disabled and by several escorts, including volunteers. Group activities and their applicability are tested before large-scale interventions begin. Potentially experienced disadvantages, injuries, hospitalizations of the subjects will be monitored throughout the group activity and 9 months after.

You can ask researcher Laura Rautiainen or research doctor Kaisu Pitkälä in more detail about possible effects and harms.

Insurance coverage and compensation of the research subjects

If your close relative suffers personal injury as a result of participating in the study, you can apply for compensation. For personal injury, you can apply for compensation from the research center's patient insurance. Kaisu Pitkälä provides more information about the insurance and applying for it (contact information below).

There is no remuneration for participating in this study. Participating in the nature-oriented Ystäväpiiri group, which is part of the study, is free for your close family members.

Personal data processing and data confidentiality (briefly)

In this study, Finnish research and personal data protection legislation is applied. Researchers and other research personnel are committed to following good scientific practice and research ethical guidelines. A more detailed description of the research's right to information basis is at the end of this release.

The personal data of your next of kin is processed for scientific research purposes. The information and research results collected about him are treated confidentially as required by legislation. All entities and persons handling the information of your immediate family are bound by confidentiality. You can find more information about personal data processing and rights at the end of this announcement.

Additional information and contact persons

If you have any questions about the study, you can contact the researcher. You can talk to the researcher about any adverse effects that may have occurred during the study and other things that concern you.

Investigator:
Laura Rautiainen
TtM, Dissertation researcher
Researcher's telephone number and e-mail:
Tel. 050 317 2562
laura.rautiainen@vtkl.fi

The research is responsible for:
Kaisu Pitkälä
LKT, general medicine. prof. (emerita),
HY- Department of General Medicine and Primary Health Care
tel. 050-3385546
kaisu.pitkala@helsinki.fi

5/1/2022

DESCRIPTION OF THE PROCESSING OF PERSONAL DATA IN THE RESEARCH AND THE RELATED RIGHTS OF THE RESEARCHER

Registrar

The registry keeper in the study is HY, which is responsible for the legality of the processing of personal data in connection with the study.

Only personal data necessary for the purpose of the study are stored in the research register. Data collection is based on a research plan.

Basis for processing personal data

In this study, it is a general interest in other scientific research:
The basis for processing personal data for the purpose of scientific research in the public interest is Articles 6.1.e and 9.2.j of the Data Protection Regulation, which is regulated in more detail in the law on medical research (L 488/1999).

Section 6 subsection 2 of the Data Protection Act applies when processing the personal data of your next of kin in research.

Processing of personal data

In the study, the personal data of your next of kin will be processed only by the persons appointed to the research group, whose job duties include processing them.

Only personal data necessary for the purpose of the study are stored in the research register. The identity of the research subjects is known only to the research staff, who are bound by confidentiality. All information collected about your next of kin in the study is processed coded after the data collection, so his or her information cannot be identified from research results, reports or publications related to the study. Coding the data means that your next of kin's name and social security number are removed and replaced with a unique code. After this, information about him cannot be identified without a code key, which is kept by the person responsible for the study. Persons outside the study do not have access to the code key. The research results are analyzed coded.

Where data is collected

The study collects personal information about your next of kin from the following sources: a letter survey at the beginning, individual interviews conducted by the researcher before and after the group, a group interview after the group, the group leaders' notes about the groups, and the researcher's observations in the group from two group sessions. The interviews conducted during the research and the observations carried out in the group will be recorded.

Transfer of personal data

In this study, the research data will be transferred without identifying information to the EU joint RECETAS project for analysis. The personal data of your immediate family will not be disclosed to other parties and will be processed for scientific research purposes. The data of your immediate family will not be transferred outside the EU and the European Economic Area (EEA).

Storage of personal data

The retention period of personal data is regulated by legislation and good clinical research practice. HY is responsible for the storage of your close relatives' personal data.

This study is an observational interview study in nature. Personal data will be destroyed no later than 10 years after the end of the research. In order to verify the accuracy of the research results, it is recommended to keep the data for a predetermined period of time after the end of the research.

Subject's rights

You have the right to receive information about the processing of personal data of your next of kin and to request the restriction of the processing of personal data. You also have the right to check his information and request that it be corrected or completed if, for example, you notice an error in it or that it is incomplete or inaccurate. You also have the right to object to the processing of the personal data of your next of kin.

However, in the context of scientific research, these rights can be limited. The law may oblige the data controller to keep your research data for a certain period of time, regardless of the data subject's rights. The law allows exceptions to the data subject's rights when it is necessary to ensure the safety of scientific research results and research subjects. In this study, these exceptions do not apply.

You can inquire at any time whether we process the personal data of your next of kin and demand a justification for the processing. You can also inquire about where we got his information from and where his information has been transferred. You have the right to receive the information free of charge and within a reasonable time (within one month of the request). If your Data Request is very extensive or for some other justified reason the collection of data is particularly time-consuming, the deadline can be extended by a maximum of two (2) months. You will be notified of the extension of the deadline and the reason.

In matters of data protection, we recommend contacting the data protection officer of the research registrar:

University of Helsinki data protection group
University of Helsinki
Address: PL 3 (Fabianinkatu 33), 00014 University of Helsinki **data protection@helsinki.fi**

You have the right to file a complaint, especially with the supervisory authority corresponding to the location of your next of kin's permanent residence, if you consider that the processing of personal data violates the EU General Data Protection Regulation (EU) 2016/679 or the Data Protection Act (1050/2018). In Finland, the supervisory authority is the Data Protection Commissioner.

Office of the Data Protection Commissioner, Lintulahdenkuja 4, 00530 Helsinki, PO Box 800, 00531 Helsinki
Telephone exchange: 029 566 6700, E-mail (register office):data protection@om.fi

CONSENT OF THE SUBJECT'S CLOSE RELATIVE TO PARTICIPATION IN THE RESEARCH

Elderly people's experiences of loneliness and the importance of nature in assisted living

Helsinki, researcher Laura Rautiainen

My close relatives[subject's name]has been asked to participate in the scientific research mentioned above, the purpose of which is to find out the loneliness experienced by the elderly living in a service home and the importance of nature experiences for their well-being. The aim of the study is to find out what kind of loneliness the subjects experience in a service home, how the subjects feel that loneliness affects their well-being, and in what ways, in their opinion, loneliness can be prevented, alleviated and reduced through nature experiences. The interviews conducted during the research and the researcher's visits to the group are recorded.

I have read and understood the research bulletin I received and I give my consent to the research according to it. I have received sufficient information from the bulletin about the study and the collection, processing and disclosure of data to be carried out in connection with it. The contents of the release have also been told to me orally and I have received sufficient answers to all my questions regarding the research. The information was provided by researcher Laura Rautiainen date].

I have had enough time to consider my next of kin's participation in the study. I have received sufficient information about the purpose of the study and its implementation, the benefits and risks of the study, and my rights. I have not been pressured or enticed to give permission to participate in the study.

I know that the information of my next of kin will be treated confidentially and will not be disclosed to outsiders.

I understand that participation in this study is voluntary. I am aware of the fact that I have the right to deny my immediate family participation in the study. Later, if my next of kin wishes, I can also interrupt his participation in the study or withdraw my consent at any time without giving a reason, and these will not affect the treatment of my next of kin or the treatment he receives in any way.

I can stop the participation of my next of kin at any stage of the study without giving a reason. I also have the right to withdraw my consent at any time before the end of the study. I am aware that if I interrupt

research or withdraw consent, the information collected about my next of kin up to the time of suspension and withdrawal of consent can be used in the research. I know that no compensation will be paid for the expenses incurred for participating in the study.

With my signature, I confirm the participation of my next of kin in this study and my consent to voluntarily become a research subject.

____
Signature of the representative of the subject

____
Date

____
Clarification of the name of the representative under investigation

____
(Kinship) relationship with the subject

____
Date of birth of the subject representative

____
Address of the agent being investigated

____
Subject's name

____
Personal identification number of the subject

____
The address of the subject

Consent received

____
Investigator's signature

____
Date

____
Clarification of name

The original signed document remains in the researcher's archive and a copy of the signed consent is given to the subject's representative.
