# Supplementary material for: Nature-based social interventions to address loneliness among vulnerable populations: a common study protocol for three related randomized controlled trials in Barcelona, Helsinki, and Prague within the RECETAS European project
Source: BMC Public Health. 2024 Jan 13;24:172. doi: 10.1186/s12889-023-17547-x (PMC10787456; doi:10.1186/s12889-023-17547-x)
Supplement: Supplementary file 2 — Additional file 2. [file 12889_2023_17547_MOESM2_ESM.pdf]

APPENDIX 2. Organisation Informed Consent

RECETAS project information
for the institutions participating in the study

We propose to participate in the **RECETAS Project**, which is a research project that aims to reduce the feeling of loneliness in elderly people who feel lonely from the social prescription of nature (i.e., linking them to natural areas and activities that take place in nearby natural environments). The study aims to assess the effectiveness of this intervention, that is, if it really improves their solitude and quality of life.

Description

This project will allow to understand the impact of homelessness from the identification of vulnerable groups and the intervention for the improvement of homelessness in natural environments from a social intervention. To do so, it will use different methodologies. The study will consist of a pilot test, a randomized clinical trial and will use a mixed methodology (quantitative and qualitative).

This project, in the first place, identifies in each territory which are those groups most vulnerable to loneliness and what natural resources are available in the area. Secondly, taking advantage of the therapeutic potential of nature, a social intervention will be developed to improve or alleviate this feeling of loneliness. This intervention is based on the "Circle of Friends" methodology from Finland where the participants start with an individual interview and then meet in a group for 9 weeks, once a week and for two hours, to participate in group activities in nature, with the ultimate goal of reducing the feeling of loneliness and improving their quality of life.

In order to evaluate the effectiveness of the intervention, the participants will be assigned randomly, that is to say, to a random, intervention group or a control group. The first group will do the "Cercle d'Amics" intervention explained in the previous paragraph. The second group will receive information, provided by professionals, on the activities that can be carried out in nature. At the end of the study, and in order to give continuity to the intervention in the areas where the project will be developed, it is planned to offer training to the participating social and health organizations so that they can incorporate it into their practice. In this way, and

provided that the entities decide to offer the intervention, the people who are part of the control group may request to participate in the intervention.

Thus, the impact of the project will be long term, in two ways. The first is from the effectiveness of the intervention itself in improving the feeling of solitude. The second is from the incorporation of the social prescription of nature in society and, specifically, in the national health system and the territorial entities.

The **RECETAS Project** is funded by the European Union through a Horizon 2020 grant with reference number H2020-SC1-BHC-2018-2020 - Project 945095. You can find the information at the following link: https://recetasproject.eu/

The **project** is carried out cooperatively by national and international professionals and organizations with an extraordinary research background in health, nutrition, physical and mental health, and nature. The project is led by the Barcelona Institute for Global Health (ISGlobal) and the principal investigator is Dr. Jill Litt. Thirteen institutions from nine different countries are participating, and the study will be carried out in six cities. In the case of Catalonia, in addition to ISGlobal, the Universitat de Vic-Universitat Central de Catalunya (UVic- UCC), the Fundació Salut i Envelliment - Universitat Autònoma de Catalunya (FSIE- UAB), and the Agència de Salut Pública de la Generalitat de Catalunya (Public Health Agency of the Generalitat de Catalunya) are participating. The study carried out in Catalonia is led by Dr. Laura Coll Planas (UVic-UCC). UVic-UCC carries out the surveys, the intervention and the qualitative evaluation. FSIE-UAB, under the responsibility of Sergi Blancafort, is in charge of identifying the territories and participants and analyzing the data. The Public Health Agency of the Generalitat de Catalunya provides support throughout the project and links areas of Catalonia with experience in social prescription to the study.

Together with the contribution of many national and international partners, this project has the support of the main actors in the sector. It is especially important to count on the participation of local social and health organizations in order to achieve a significant impact on the territory and to ensure its continuity over time. In this way, all the ingredients are provided for this to become a project with a great transforming capacity and a high social and scientific impact, both nationally and internationally.

The study complies with all ethical requirements and has the approval of the UVic-UCC Research Ethics Committee.

Implicacions

Participation in the project includes different implications that we detail below: This study requires the collection of personal data:

1. During the process of identification and recruitment of participants, we will ask for contact information (name, name and contact telephone number) of persons linked to your organization in order to apply the eligibility criteria and, if they complete them, to offer their participation in the study, which will be made effective by signing the informed consent, after explanation and reading of the information packet. If you do not have your own personal data transfer authorization document, we will provide you with a project-specific one to carry out this point.
2. Collection of information directly from the people participating in the study:
    a. During the quantitative fieldwork, socio-demographic, health and welfare data of the participants will be collected.
    b. In the qualitative fieldwork, individuals and professionals will be invited to participate. Both the interviews and the groups will be audio-recorded (without video), transcribed for analysis and pseudo-anonymized. If images are generated that express the experience, written permission will be requested to use these sources of information in the communication of the project.
3. Data from the computerized clinical history corresponding to the use of health resources, medication and comorbidity will be requested from the participant's Primary Care Center prior to authorization by the person by signing the informed consent form.

Avantatges

Beyond being able to contribute to science, scientific production and dissemination, the benefits and considerations foreseen by the centers are:

1. The "Nature-based Social Prescription" training will be offered to a maximum of *three professionals per intervention area,* who may be from the same or different centers,

with the objective that the knowledge remains in the territory and that the intervention can be offered to the control group and other groups, and that the impact of the project continues over time. In addition, the project will offer two trainings: one in January/February 2023 to train professionals who will be involved in dynamizing the groups of the clinical assessment on a paid basis (prior selection) and another training in 2025-26 to disseminate the knowledge acquired in the territory. It will be possible to count on the support/advice from UVic/FSIE professionals in order to implement the intervention once the project is finished.

2. Free lectures will be offered on demand on topics of expertise of the research team in the field of sunbathing, healthy skin and nature as a source of health.
3. The participation of professionals from the center in scientific communications will be considered.
4. Certificates of participation and/or collaboration in the research project, etc. are issued by the center or by professionals involved in a way that is useful for their professional career.
5. A session of return of the results of the project will be proposed to the specific center or jointly with the centers of the same area of intervention.

Data protection

According to the Organic Law 3/2018, of December 5, on the protection of personal data and guarantee of digital rights, all data obtained will be analyzed and used only for the scientific objectives of the study. The data will be stored in secure servers of UVic-UCC and ISGLOBAL, with access restricted by password and limited to the research team. The identification of organizations and participants will be encrypted and pseudo-anonymized, protecting the identification codes. In no case will any personal data be disclosed.

To confirm participation, it is necessary to fill out the INFORMED CONSENT for the participation of the institution in the RECETAS Project and send it to Laura Coll at: laura.coll@uvic.cat or to Sergi Blancafort at sergi.blancafort@uab.cat.

Subsequently, we will propose a **day to hold a face-to-face or online meeting** to present the project in more detail, clarify any doubts you may have regarding your participation and

their implications. On our part we guarantee, at all times, the security of the study, with the protection of data of all participants.

Thank you very much,

Dr. Laura Coll PlanasDr. Sergi Blancafort Alias

INFORMED CONSENT

for the institution's participation in the RECETAS project

Jo, (name and cognoms)

---

as a (càrrec, per exemple: manager, director or coordinator)

---

of the organization (name of the institution)

I agree to participate in the **RECETAS Project.**

DECLARE THAT

I have received information about the **RECETAS Project**, of which I have been given the information attached to this consent and for which I request the participation of the institution I represent.

I have understood its meaning, my doubts have been solved and the actions derived from it have been explained to me.

I have been informed of all the aspects related to the confidentiality and protection of personal data involved in the project, and the guarantees given in compliance with the Organic Law 3/2018, of December 5, on the protection of personal data and guarantee of digital rights and the General Regulation (EU) 2016/679, of April 27, 2016, on data protection and complementary regulations.

The participation of the institution in the project is completely voluntary and I have the right to withdraw at any time, revoking this consent, without this withdrawal having any negative influence on the institution.

In case of withdrawal, I have the right to have my data deleted from the study archive. I also renounce to any economic, academic or any other kind of benefit that may derive from the project or its results.

For all this,

I GIVE MY CONSENT:

That the institution I represent participates in the **RECETAS Project:**

1. That the research team of the **RECETAS Project** and Laura Coll Planas, as principal investigator, can manage the institution's data and disseminate the information generated by the project. It is guaranteed that my identity and privacy of the institution will be preserved at all times, with the guarantees established in the Organic Law 3/2018, of December 5, 2018, on the Protection of Personal Data and guarantee of the privacy of personal data.

digital rights, and the General Regulation (EU) 2016/679, of 27 April 2016, on data protection and complementary regulations.

2. That the **RECETAS Project** team keeps all the records made on the institution in electronic support, with the guarantees and in the terms legally provided, in the Organic Law 3/2018, of December 5, on the protection of personal data and guarantee of digital rights; and that in the absence of legal provision, for the time necessary to fulfill the functions of the project for which the data had to be collected.

[ciutat] ____ , on [date] ____ / ____ / 202 ___

Signature of the person responsible for the institution

Signature of the researcher
