# Supplementary material for: Nature-based social interventions to address loneliness among vulnerable populations: a common study protocol for three related randomized controlled trials in Barcelona, Helsinki, and Prague within the RECETAS European project
Source: BMC Public Health. 2024 Jan 13;24:172. doi: 10.1186/s12889-023-17547-x (PMC10787456; doi:10.1186/s12889-023-17547-x)
Supplement: Supplementary file 3 — Additional file 3. [file 12889_2023_17547_MOESM3_ESM.pdf]

This project has received funding from the European Union's Horizon 2020 research and innovation programme under grant agreement No 945095.

Informed consent to participate in the RECETAS research project

Subject: Center for the study of longevity and long-term care, Faculty of Humanities, UK
Contact person: Mgr. Vladimíra Dostálová, Ph.D. e-mail: vladka.dostalova@seznam.cz

Investigators of the RECETAS research project
(researchers): doc. MD Iva Holmerová, Ph.D.
M.Sc. Alžběta Bártová, Ph.D. M.Sc.
Vladimíra Dostálová, Ph.D. Bc.
Barbora Holubová
M.Sc. Michal Karl'a, Ph.D.

Dear Madam, dear Sir,
We are contacting you with a request to participate in a research project carried out by the Faculty of Humanities of Charles University, which is called RECETAS[1].

The aim of the project is to obtain data on the usefulness of recommending group outdoor activities and activities in nature for alleviating the feeling of loneliness. The information we obtain thanks to your participation in the RECETAS project will be used for scientific purposes. They will be presented to the professional public or published anonymously in the professional press. Your participation will significantly contribute to the expansion of knowledge about the possibilities of recommending social activities in nature in connection with reducing the feeling of loneliness.

Your participation in the project, which will last one year (twelve months), will consist of:

1. In participating in questionnaire surveys, which will be four in total. The first questionnaire survey will be carried out at the beginning of your involvement in the research project and after three, six and twelve months of your participation. Each survey will take about 40 minutes. The questionnaire is completely anonymous.
2. In participating in ten group meetings. The goal of the first meeting will be a joint choice of activities outdoors and in nature, and then the implementation of these activities once a week (for a period of nine weeks).
3. In conducting an individual interview, which will be conducted at the end of your participation in this project.

Before each questionnaire survey and individual interview, you will be asked to give informed consent. Sample informed consent forms are attached to this document.

Your personal data, which you provide to members of the research team as part of the implementation of the RECETAS project, will not be published anywhere and will be handled in accordance with Act No. 110/2019 Coll., on

[1]RECETAS – Re-imagining Environments for Connection and Engagement: Testing Actions for Social Prescribing in Natural Spaces

RECETAS

This project has received funding from the European Union's Horizon 2020 research and innovation programme under grant agreement No 945095.

processing of personal data, as amended, Act No. 89/2010 Coll., Civil Code, as amended and in accordance with Regulation (EU) 2016/679, on the protection of natural persons in connection with the processing of personal data and on the free movement of such data (GDPR).

Your participation in the RECETAS project is not burdened with any risk. If any activity within the project would be uncomfortable for you, you do not have to participate in it, you are not obliged to answer questions that would be uncomfortable for you.

With respect and thanks, RECETAS team

Affidavit

a. I declare that I have been informed that according to the above legal regulations on the processing of personal data I have the right to:

- revoke the given consent at any time, for each of the above purposes separately;
- request information about what my personal data is being processed;
- request correction or addition of my personal data;
- request deletion of personal data for which there is no longer a reason for processing;
- request restriction of processing of data that is inaccurate, incomplete or for which the reason for processing has ceased to exist, but I do not agree to their deletion;
- request the transfer of processed data;
- to object to the processing of my personal data for direct marketing, including related profiling;
- not be subject to automated individual decision-making, including profiling;
- I have the right to receive a response to my request without undue delay, in any case within one month of receipt of the request by the administrator.

The following contact (phone number, email address) for a member of the RECETAS research team can be used to contact the administrator regarding the processing of personal data:
Iva Kolářová, phone: 777 888 426; e-mail: recetas@gerontocentrum.cz

b. I confirm that I have read and understood this informed consent regarding the above research.

c. I declare that I have been instructed about the possibility of asking questions. I also declare that I understand and take note of all the facts and information provided above. I have no further questions or ambiguities and express my express free consent to participate in the research project.

d. I declare that I am fully competent for legal actions and as such I declare that I am informed of the fact that my participation in the project is voluntary and I am entitled to withdraw from the research project at any time.

e. I declare that I acknowledge the information contained in this informed consent and agree to the processing of my personal and sensitive data to the extent and in the manner and for the purpose specified in this informed consent.

This consent is written in two copies with the nature of the original, with one copy received by the research participant and one copy received by the entity implementing the research project.

Party research:	First and last name (printable)	Signature	Place	Date
Researcher:	First and last name (printable)	Signature	Place	Date

Appendix 1. Consent to taking photos, sound and video recordings Appendix 2. Sample consent to participate in a questionnaire survey
Appendix 3. Sample of consent to conduct individual interviews
